# Distribution of molecular subtypes in a Colombian cohort of patients with acute lymphoblastic leukemia using whole-transcriptome analysis

**DOI:** 10.3389/fonc.2026.1957558

**Published:** 2026-09-23

**Authors:** Edward Perez-Arismendi, Natalia Gomez-Lopera, Juan Camilo Villada, Diana Moreno-Garcia, Federico Arroyave-Ossa, Lina Maria Quiroz, Natalia Valencia-Zuluaga, Javier Munoz-Martinez, Alexandra Restrepo-Rincon, Andres Garces-Arias, Gonzalo Vasquez-Palacio

**Affiliations:** 1 Grupo de Investigación Genética Médica, Facultad de Medicina, Universidad de Antioquia, Medellín, Colombia; 2 Servicio de Oncohematología Pediátrica y Trasplante de Médula Ósea, Hospital San Vicente Fundación, Medellín, Colombia; 3 Laboratorio especializado, Clínica Colsanitas, Bogotá, Colombia; 4 Servicio de Oncología Pediátrica y Trasplante de Médula Ósea, Departamento de Cancerología, Hospital Pablo Tobón Uribe, Medellín, Colombia; 5 Unidad de Oncología, Hospital Alma Máter de Antioquia, Medellín, Colombia

**Keywords:** acute lymphoblastic leukemia, differential expression analysis, gene fusions, molecular subtypes, Ph-like ALL, RNA-seq, single nucleotide variants, whole-transcriptome sequencing

## Abstract

**Introduction:**

Acute lymphoblastic leukemia (ALL) is a common pediatric malignancy and shows marked genetic heterogeneity. Transcriptomic profiling may improve molecular diagnosis and risk stratification, particularly in admixed Latin American populations.

**Methods:**

We characterized the transcriptomic landscape of pediatric ALL in 40 Colombian patients aged <20 years using whole-transcriptome RNA sequencing of diagnostic bone marrow or peripheral blood samples. Bioinformatic analyses enabled molecular subtype assignment, fusion detection, differential expression analysis, and variant calling.

**Results:**

The median age was 8 years, and 8 patients had measurable residual disease at the end of induction. The most frequent subtypes were *ETV6::RUNX1* and Ph-like, each representing 15% of cases. Fusions, including *MEF2D::HNRNPUL1* and *SEPTIN9::ABL1*, were identified. Differential expression analysis revealed 3,864 genes distinguishing ALL from non-leukemic controls, with enrichment of hematologic malignancy, B-cell differentiation, immune, and inflammatory pathways.

**Conclusions:**

Molecular classification refined conventional risk stratification in 10% of patients, supporting the potential clinical utility of transcriptomic profiling in pediatric ALL.

## 1 Introduction

Acute lymphoblastic leukemia (ALL) is a heterogeneous malignancy characterized by the uncontrolled proliferation of lymphoid precursors in the bone marrow and/or peripheral blood. It is the most common childhood cancer, accounting for approximately 25% of pediatric malignancies (1, 2). Although cure rates in high-income countries can reach up to 92%, outcomes in Latin America remain considerably lower, with reported cure rates of approximately 70% and 53.8% in Colombia (3–6). In Latin America, the incidence of childhood leukemia has been reported at approximately 4.4 cases per 100,000 population, while in Colombia, it approaches 5 cases per 100,000 population (7). These disparities have been associated with barriers to timely diagnosis and treatment, limited access to advanced diagnostic technologies, and limited implementation of molecular profiling in routine clinical practice (3–5). In addition, the genetic diversity of Latin American populations may influence the distribution of molecular subtypes and the clinical course of the disease (8, 9).

In routine clinical practice in Colombia, genetic alterations are mainly detected using conventional cytogenetics, fluorescence *in situ* hybridization (FISH), and qPCR. Although these techniques are useful, they have limitations in identifying clinically relevant molecular subtypes, thereby restricting prognostic stratification (10). In this context, whole-transcriptome sequencing (RNA-seq) has emerged as a key approach for defining molecular subtypes by simultaneously identifying gene fusions, expression profiles, single-nucleotide variants, and cryptic alterations not detectable by conventional cytogenetics (11, 12).

Transcriptomic studies in ALL have enabled the identification of molecular subgroups defined by gene expression profiles and recurrent genetic alterations, including *ETV6::RUNX1*, *hyperdiploidy*, *DUX4*-rearranged, *ZNF384*-rearranged, *KMT2A*-rearranged, *MEF2D*-rearranged, and Ph-like ALL, each associated with distinct biological and prognostic features (13–15). These advances have reshaped the biological understanding of B-cell ALL and contributed to the incorporation of new molecular entities into the fifth edition of the World Health Organization (WHO) classification (16). Clinically relevant alterations involving *PAX5* and *IKZF1*, including the *IKZF1*plus genotype, have also been associated with poorer survival and a higher risk of relapse (13).

In Latin America, although transcriptomic studies based on RNA sequencing in pediatric ALL remain limited, recent experiences in Argentina, Brazil, and Mexico have demonstrated RNA-seq’s potential to improve molecular characterization. In Argentina, whole-transcriptome sequencing enabled molecular subtype assignment in more than 90% of cases, revealing a heterogeneous distribution that included hyperdiploidy, *ETV6::RUNX1*, Ph-like ALL, and less frequent subtypes such as *MEF2D*-rearranged ALL. In Brazil, targeted RNA-seq reclassified approximately two-thirds of cases previously categorized as B-other, identifying both classical subgroups and alterations associated with high biological risk. In Mexico, transcriptomic analysis confirmed recurrent fusions and identified subtypes including hyperdiploidy (~27%), *DUX4*-rearranged ALL (~13%), and Ph-like ALL (~9%) (17–19). However, RNA-seq-based transcriptomic studies in pediatric ALL have not yet been reported in Colombia. Generating local evidence is essential for describing the distribution of molecular subtypes, comparing it with other populations, and strengthening prognostic stratification in the national context. Therefore, this study aimed to characterize the transcriptomic landscape of a cohort of Colombian patients with ALL using RNA-seq.

## 2 Materials and methods

### 2.1 Patients and samples

This observational, exploratory, cross-sectional analytical study included Colombian patients under 20 years of age with newly diagnosed acute lymphoblastic leukemia (ALL). Participants were recruited consecutively by convenience sampling at three institutions in Medellín, Colombia (Hospital San Vicente Fundación, Hospital Pablo Tobón Uribe, and Hospital Alma Máter de Antioquia) until the target sample size was reached. A total of 49 patients were initially assessed; of these, nine were excluded (six due to poor RNA quality and three due to insufficient genetic material for downstream analyses), yielding a final sample of 40 patients. Samples were collected at diagnosis from peripheral blood (n = 4) or bone marrow (n = 36); peripheral blood samples showed a blast percentage broadly comparable to that of bone marrow samples (>80%), which may have helped reduce, although not eliminate, potential bias in differential expression profiles related to differences in cellularity between sample types. Patients were treated according to the institutional protocols in use during the study period, including ALL-IC BFM 2009, ALL-IC BFM 2022, CALGB 10403, and GRAALL 2005.

Sociodemographic and clinical data, including age, sex, cytogenetic and molecular test results, measurable residual disease (MRD), and treatment response, were obtained from medical records. MRD was assessed by flow cytometry as part of routine clinical care according to the corresponding treatment protocol. For patients treated under ALL-IC BFM protocols, MRD was evaluated at mid-induction and at the end of induction, whereas for patients treated according to CALGB 10403 or GRAALL 2005, MRD was evaluated at the end of induction. Additionally, five non-leukemic bone marrow samples were included as controls, obtained from patients evaluated for suspected conditions other than leukemia in whom the diagnosis was subsequently ruled out.

### 2.2 RNA extraction and sequencing

Total RNA extraction and ribosomal RNA (rRNA) depletion were performed, followed by cDNA library preparation using the QIAseq FastSelect rRNA/Globin HMR system (Qiagen) in combination with the NEBNext Ultra II Directional RNA Library Prep Kit (New England Biolabs), according to the manufacturers’ instructions. Sequencing was performed in a paired-end 2 × 150 bp format on the NovaSeq X Plus platform (Illumina), generating approximately 60 million paired-end reads per sample. This workflow was performed by Admera Health (South Plainfield, NJ, USA).

### 2.3 Bioinformatic analysis

#### 2.3.1 Quality control and sequence alignment

The quality of raw FASTQ reads was assessed using FastQC v0.11.9 (20) based on standard quality metrics. Overall, the reads showed high sequencing quality. Adapter trimming and removal of low-quality bases (Q20) were performed using Trimmomatic v0.39, and post-trimming quality was reassessed with FastQC. Filtered reads were aligned to the GRCh38 reference genome (Ensembl) using STAR v2.7.10b (21), and SAM/BAM files were processed and validated with SAMtools v1.10 (22). Sequencing quality-control results are presented in **Supplementary Figure 1**, which shows the per-base sequence quality profiles, and **Supplementary Table 1**, which provides per-sample metrics including read number, read length, alignment rate, coverage uniformity, and library strandedness.

#### 2.3.2 Differential expression analysis and molecular subtype prediction

Gene-level quantification was performed using featureCounts v2.0.2 (23), and differential expression analysis was subsequently conducted in RStudio v2024.12.1563 using DESeq2 (24). To ensure data quality, raw counts were filtered to retain genes with a total count sum ≥10 across all samples, after which data were transformed using variance stabilizing transformation (VST). The resulting VST-transformed data were then used to generate heatmaps based on unsupervised hierarchical clustering, with gene expression scaled per gene and samples annotated by biological condition and molecular subtype. Potential batch effects associated with the four sample-processing submissions were assessed using PERMANOVA on the transformed expression data. Based on the relatively limited proportion of expression variability attributable to batch, batch was not included as a covariate in the final DESeq2 differential-expression model. For significance assessment, genes with an adjusted p-value <0.05 and FC ≥2 were considered significantly differentially expressed.

Finally, molecular subtypes were primarily assigned using MD-ALL (25) and independently assessed using ALLCatchR (26). The two classifiers showed concordance in 38 of 40 evaluable cases (95%), considering cases concordant when the ALLCatchR prediction matched at least one of the subtypes proposed by MD-ALL. Discordant cases were reviewed considering gene fusion, cytogenetic, and other molecular findings. Per-sample predictions from each classifier and the final assigned subtype are detailed in **Supplementary Table 2**.

#### 2.3.3 Fusion gene and single-nucleotide variant detection

Fusion genes were detected using the DRAGEN RNA Pipeline v4.3.13 in BaseSpace (27). Single-nucleotide variants were identified using GATK v4.2.0.0, including SplitNCigarReads, Mutect2, and FilterMutectCalls (28), and annotated with OpenCRAVAT (29), integrating multiple pathogenicity and cancer databases. Quality filters were applied using a read depth ≥30 and an alternate allele depth ≥10. Clinical interpretation was performed using Franklin (30) and VarSome (31). Because matched germline DNA was not available, variants were considered RNA-seq-detected variants and their somatic origin could not be definitively established.

### 2.4 Statistical analysis

Clinical, sociodemographic, and molecular characteristics were summarized using descriptive statistics. Specifically, categorical variables were reported as absolute and relative frequencies, while continuous variables were presented as medians and interquartile ranges (IQR). To assess associations, Fisher’s exact test or the Mann–Whitney U test was applied, as appropriate. A p-value < 0.05 was considered statistically significant, and all analyses were performed in Jamovi v2.4 (32).

## 3 Results

### 3.1 Clinical characteristics of the cohort

The median age was 8 years (IQR: 5–11.3), with a slight female predominance (52.5%). B-cell ALL was the most frequent immunophenotypic subtype (90%), with a median leukocyte count at diagnosis of 14.6 × 10^9^/L (IQR: 6.8–34.3). Intermediate-risk disease was the most frequent clinical risk category (45%), followed by high-risk disease (40%). Favorable steroid response on day 8 was observed in 85% of cases, central nervous system involvement in 32.5%, and negative MRD at the end of induction in 79.5% (**Table 1**).

**Table 1:** Clinical and sociodemographic characteristics of the study cohort.

Characteristic	n (%)	Median (IQR)
Age (years)		8 (5–11.3)
Sex		
-Male	19 (47.5%)	
-Female	21 (52.5%)	
Immunophenotypic subtype		
-B-cell ALL	36 (90%)	
-T-ALL	4 (10%)	
Leukocyte count at diagnosis (×10^9^/L)		14.6 (6.8–34.3)
Clinical risk group		
-High risk	16 (40%)	
-Intermediate risk	18 (45%)	
-Standard risk	6 (15%)	
Steroid response on day 8		
-Good	34 (85%)	
-Poor	6 (15%)	
CNS involvement		
-Positive	13 (32.5%)	
-Negative	27 (67.5%)	
End-of-induction MRD		
-Negative	17 (42.5%)	
-Positive	23 (57.5%)	
MRD at the end of induction^†^		
-Negative	31 (79.5%)	
-Positive	8 (20.5%)	
Relapse*		
-Yes	2 (5%)	
-No	38 (95%)	
Death*		
-Yes	6 (15%)	
-No	34 (85%)	

Clinical and sociodemographic characteristics of the 40 patients with acute lymphoblastic leukemia included in the study are summarized. Categorical variables are presented as n (%), and continuous variables as median and interquartile range (IQR). End-of-induction MRD was evaluated in 39 patients; one patient died before this assessment could be performed. Relapse and death outcomes correspond to the clinical status documented at the last available follow-up. Not all patients had completed treatment at the time of analysis; the most recently enrolled patient was in the long-term maintenance phase.

ALL, acute lymphoblastic leukemia; B-cell ALL, B-cell acute lymphoblastic leukemia; T-ALL, T-cell acute lymphoblastic leukemia; CNS, central nervous system; MRD, measurable residual disease; IQR, interquartile range.

^†^MRD at the end of induction was available for 39 patients; one patient died before this assessment could be performed.

*Relapse and death outcomes correspond to the clinical status documented at the last available follow-up. Not all patients had completed treatment at the time of analysis; the most recently enrolled patient was in the long-term maintenance phase.

### 3.2 Cytogenetic findings

Cytogenetic analysis showed a normal karyotype in 40% of patients, whereas the remaining cases displayed structural (27.5%), numerical (17.5%), or complex abnormalities (15%). Identified alterations included translocations such as t(1;19) and t(9;22), the latter corresponding to Ph-positive ALL, as well as cases of hyperdiploidy. Monosomies, partial deletions, and highly complex karyotypes with multiple rearrangements, chromosomal gains and losses, and marker chromosomes were also observed, suggesting genomic instability.

Among cases with a normal karyotype, transcriptomic analysis identified alterations not detected by conventional cytogenetics, including *ETV6::RUNX1* fusions*, ETV6::RUNX1*-like expression profiles, hyperdiploid subtypes, and Ph-like cases. In addition, a *KMT2A::AFF1* fusion was confirmed by both FISH and RNA-seq.

According to NCCN guidelines (33), 40% of patients harbored cytogenetic abnormalities associated with standard risk, whereas 20% had high-risk cytogenetic alterations. Detailed cytogenetic findings are provided in **Supplementary Table 3**.

### 3.3 Molecular subtype distribution

Molecular subtypes were identified in all patients included in the study (**Figure 1**). In most cases, subtype assignment was concordant between the two transcriptome-based classifiers, MD-ALL and ALLCatchR; discordant cases were resolved by integrating classifier confidence, prediction stability, and available complementary molecular and cytogenetic findings. The most frequent subtype was hyperdiploidy (n = 8, 20.0%; 95% CI, 10.5–34.8%), followed by *ETV6::RUNX1* (n = 6, 15.0%; 95% CI, 7.1–29.1%) and Ph-like ALL (n = 6, 15.0%; 95% CI, 7.1–29.1%), and *TCF3::PBX1* (n = 3, 7.5%; 95% CI, 2.6–19.9%). Less frequent subtypes included *ETV6::RUNX1*-like, iAMP21, *MEF2D*, and *DUX4*(n = 2, 5.0% each) whereas Ph-positive ALL, *ZEB2/CEBP*, *PAX5*alt, and *KMT2A* were each identified in single cases (n = 1, 2.5% each).

**Figure 1:**
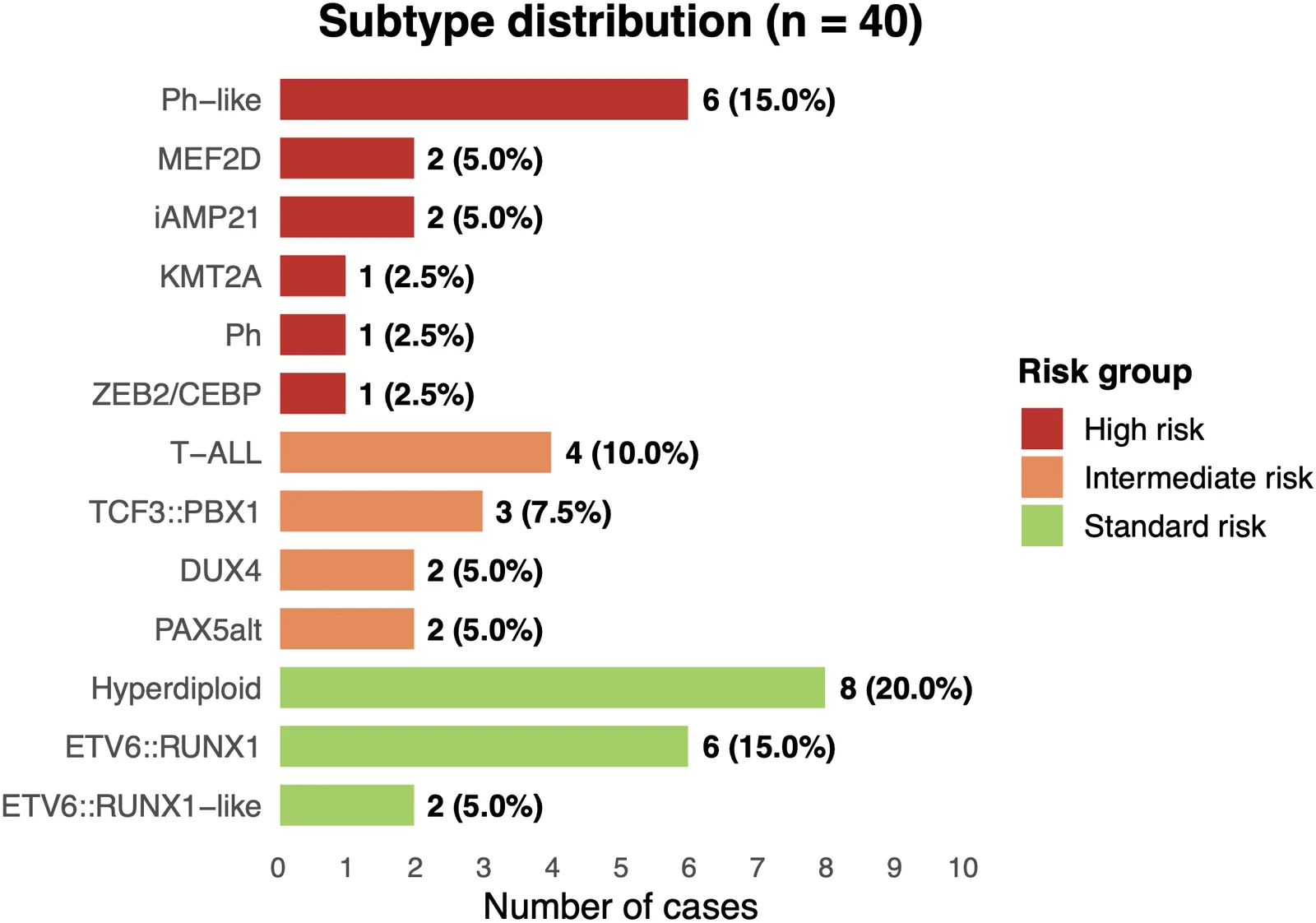
Distribution of molecular subtypes in the study cohort. Absolute and relative frequencies of the molecular subtypes identified among the 40 patients included in the study are shown. Hyperdiploidy was the most frequent subtype (n = 8; 20%), followed by ETV6::RUNX1 and Ph-like ALL (n = 6; 15% each). Colors indicate the clinical risk group associated with each subtype: high risk in red, intermediate risk in orange, and standard risk in green.

Integration of molecular classification modified the initial clinical risk stratification in 10% of the patients evaluated. Specifically, two patients initially classified as intermediate risk were reclassified as high risk after identification of molecular subtypes associated with adverse prognosis, namely Ph-like ALL and *MEF2D*. Similarly, two patients initially classified as standard risk were reassigned to higher-risk categories: one to intermediate risk, associated with the *PAX5*alt subtype, and one to high risk, corresponding to iAMP21. No patient was reclassified into a lower-risk category.

### 3.4 Gene fusions

A total of 14 fusion events were detected, corresponding to eight distinct gene fusions. The most frequent fusion was *ETV6::RUNX1* (n = 6; 42.86%), followed by *TCF3::PBX1* (n = 3; 21.43%). The remaining fusions were each identified in a single case (n = 1; 7.14%): *PAX5::P2RY8*, *SEPTIN9::ABL1*, *MEF2D::HNRNPUL1*, *MEF2D::BCL9*, and *KMT2A::AFF1*. Together, *ETV6::RUNX1* and *TCF3::PBX1* accounted for 60% of all detected fusion events. **Figure 2A** summarizes the chromosomal positions of the breakpoints and the connections between the chromosomes involved, highlighting the diversity of *MEF2D* fusion partners, including *HNRNPUL1* and *BCL9*. **Figure 2B** shows the distribution of fusion events. Detailed technical information for each RNA-seq-detected fusion event is provided in **Supplementary Table 4**.

**Figure 2:**
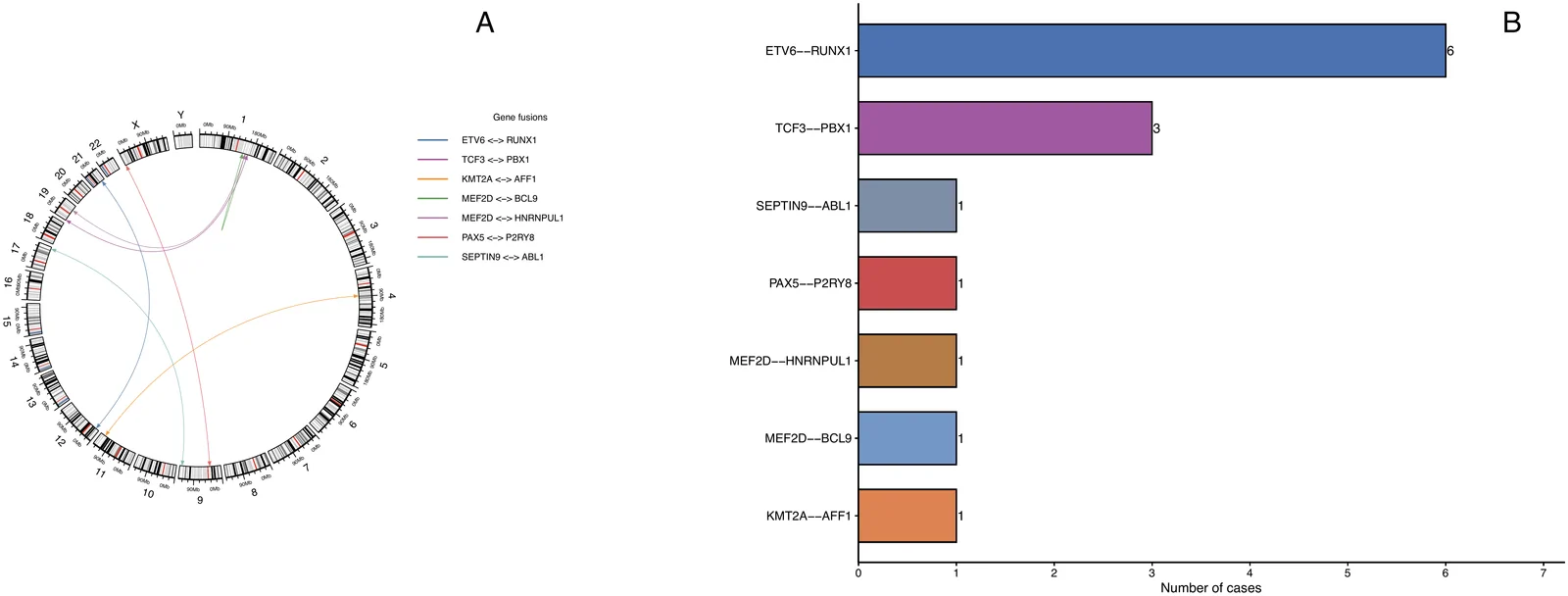
Distribution and chromosomal localization of gene fusions identified by RNA-seq. The figure summarizes fusion events detected in patients with acute lymphoblastic leukemia. **(A)** Circos plot showing the chromosomal location of the genes involved and the corresponding fusion connections. **(B)** Absolute frequency of gene fusions identified in the cohort. ETV6::RUNX1 was the most frequent fusion (n = 6), followed by TCF3::PBX1 (n = 3), whereas the remaining fusions were each detected in single cases.

### 3.5 Association between molecular subtype-associated risk and clinical variables

The association between molecular subtype-associated risk and clinical and sociodemographic variables was evaluated. A significant association with age group was observed (Fisher’s exact test, p = 0.032), indicating differences in risk distribution across age categories. Children aged 1–5 years had the highest proportion of standard-risk cases, whereas patients aged 6–14 years showed a more heterogeneous distribution, with greater representation of intermediate- and high-risk categories. Adolescents were mainly distributed within the intermediate- and high-risk groups. Molecular subtype-associated risk was also correlated with treatment response on day 8 (p = 0.032); all standard-risk cases and most intermediate-risk cases showed a favorable response, whereas the high-risk group accounted for the highest proportion of poor responses. No significant associations were found with MRD on days 15 or 33, central nervous system involvement, or socioeconomic status.

### 3.6 Differential expression analysis

A total of 3,864 differentially expressed genes were identified between ALL samples and non-leukemic bone marrow controls, using an absolute FC ≥2 and an adjusted p-value <0.05 as significance criteria. Of these, 1,337 genes were upregulated in ALL, whereas 2,527 were downregulated relative to controls. Hierarchical clustering based on these genes revealed distinct transcriptomic patterns between both groups (**Figure 3**).

**Figure 3:**
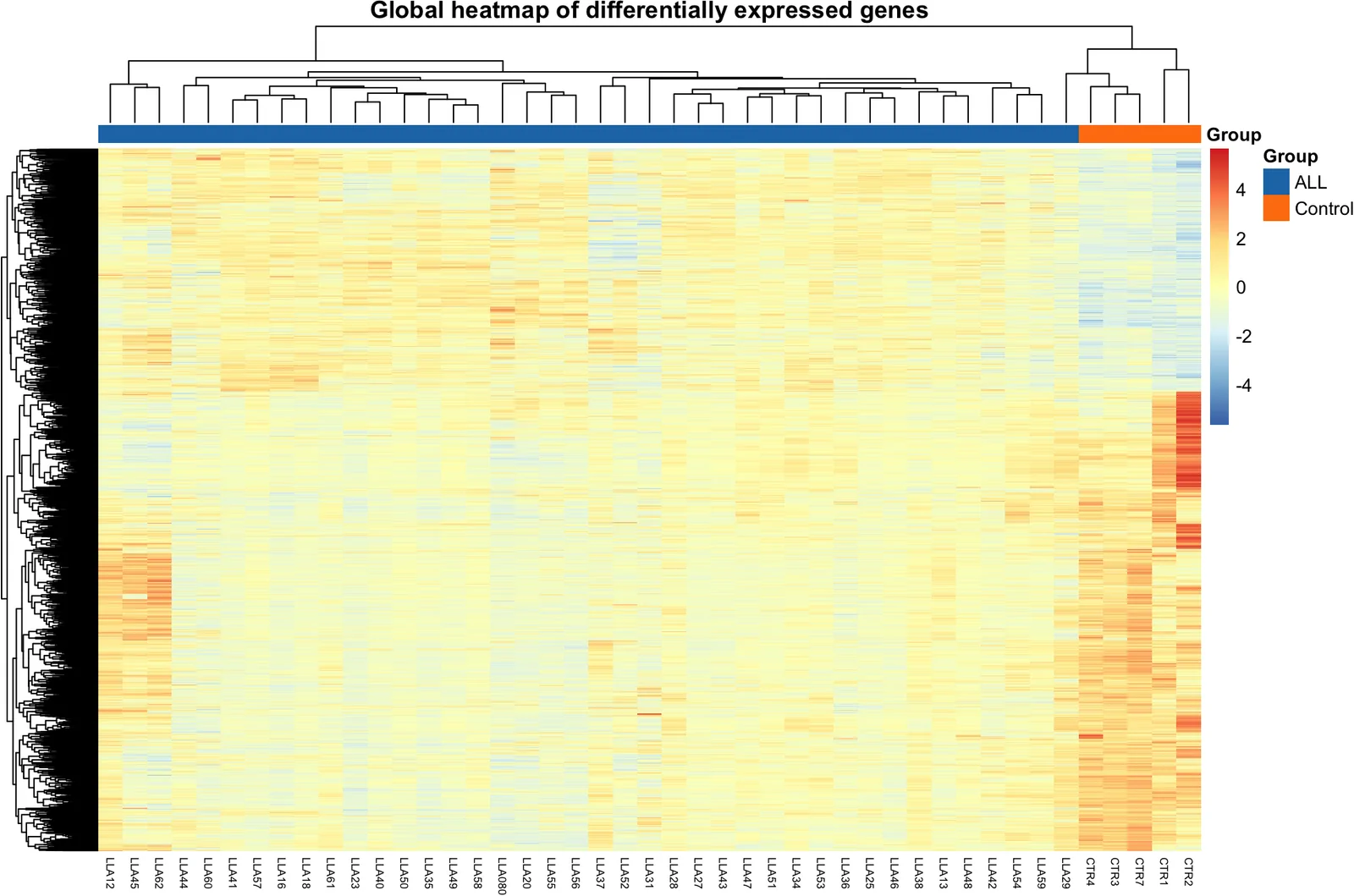
Global heatmap of differentially expressed genes between ALL samples and non-leukemic bone marrow controls. The heatmap shows 3,864 differentially expressed genes identified using an absolute FC ≥2 and adjusted p value <0.05. Rows represent genes and columns represent samples, with expression values scaled by gene. The top annotation indicates sample condition: ALL and control.

To explore the biological relevance of these transcriptional changes, functional enrichment analyses were performed separately for upregulated and downregulated genes in ALL. Upregulated genes showed significant enrichment for disease-gene association terms related to hematologic malignancies, including acute leukemia, lymphoid leukemia, hematologic cancer, and lymphoma. In contrast, genes downregulated in ALL were mainly enriched in biological processes related to immune response, inflammation, and defense mechanisms against biotic stimuli, including inflammatory response, immune system process, defense response, and regulation of immune response.

Among the differentially expressed genes, we further explored genes previously associated with ALL or lymphoid differentiation. Upregulated genes included markers of B-cell lineage and early lymphoid differentiation, such as *VPREB1, DNTT, RAG1, RAG2, PAX5, EBF1, IGLL1, BLNK, CD79A, CD19, CD79B, MME/CD10*, and *CD22*. Genes with known relevance in ALL biology, including *FLT3*, *MEF2C, TCF3, IKZF2, BCL9, AFF3, LEF1, RUNX1*, and *ABL2*, also showed higher expression in leukemic samples than in controls.

### 3.7 Single-nucleotide variants

A total of 36 variants in 24 leukemia- and cancer-related genes were identified in 15 patients from the cohort (**Figure 4**). The molecular subtypes with the highest number of variants were hyperdiploidy, *ETV6::RUNX1*-like, T-ALL, and Ph-like ALL. The most recurrently altered genes included *KMT2A*, *LEF1*, *FLT3*, *CREBBP*, *DNM2*, *SMAD4*, *EP300*, *TCF3*, and *RPL5*.

**Figure 4:**
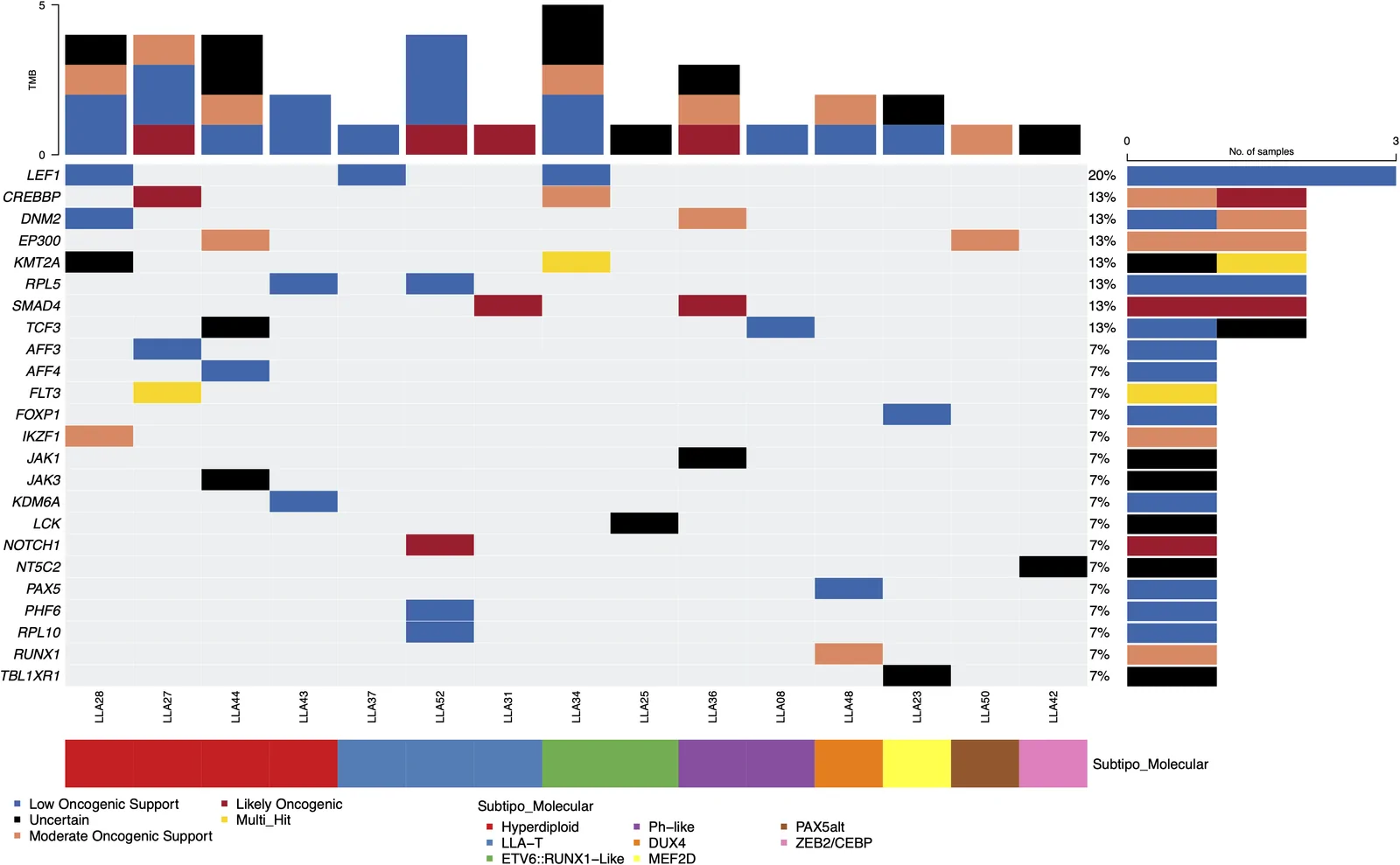
Distribution of single-nucleotide variants in ALL-associated genes. Oncoplot showing the distribution of expressed variants identified in 15 patients from the cohort. Each column represents a patient, and each row represents an altered gene. Colors indicate the variant type or functional/oncogenic classification according to the annotation performed. The upper bar summarizes the number of variants per patient, the right panel shows the alteration frequency per gene, and the lower annotation indicates the molecular subtype assigned to each patient. Variants affected genes related to cell signaling, epigenetic regulation, lymphoid differentiation, and transcriptional control, including *FLT3, JAK3, SMAD4, KMT2A, CREBBP, EP300, TCF3, LEF1*, and *RPL5*.

The clinical relevance of the detected variants was evaluated according to the AMP/ASCO/CAP framework, with Franklin and VarSome used to support annotation and interpretation. Most variants were classified as Tier 3, reflecting limited or uncertain clinical evidence. However, variants with higher levels of evidence were also identified. Two Tier 1 variants were detected in *FLT3* and *JAK3*, both in patients with hyperdiploid ALL, while Tier 2 variants were identified in *SMAD4*, including a likely oncogenic variant in a patient with Ph-like ALL. Because matched germline DNA was not available, these tier assignments reflect the potential clinical relevance of the RNA-seq-detected variants but do not establish their somatic origin.

## 4 Discussion

This study represents one of the first transcriptomic characterizations of pediatric patients with ALL in Colombia using RNA-seq. This approach enabled the identification of molecular subtypes, gene fusions, differential expression profiles, and single-nucleotide variants across the entire cohort. The most frequent molecular subtypes were hyperdiploidy (n = 8, 20.0%; 95% CI, 10.5–34.8%), *ETV6::RUNX1* (n = 6, 15.0%; 95% CI, 7.1–29.1%), and Ph-like ALL (n = 6, 15.0%; 95% CI, 7.1–29.1%), the latter being particularly relevant due to its association with high-risk disease and its limited detectability with conventional cytogenetic approaches. In addition, RNA-seq identified 14 fusion events corresponding to eight distinct gene fusions, mainly *ETV6::RUNX1* and *TCF3::PBX1*, as well as variants in 24 genes previously implicated in leukemia and cancer biology.

Cytogenetic analysis revealed marked chromosomal heterogeneity, with normal karyotypes in 40% of patients and structural, numerical, or complex abnormalities in the remaining 60%, consistent with previous reports showing that many pediatric ALL cases harbor cryptic rearrangements not detectable by conventional cytogenetics (34, 35). This was particularly evident among Ph-like cases, four of which showed a normal karyotype despite a transcriptomic profile consistent with this subtype. Similarly, *ETV6::RUNX1* fusions were identified in cases without cytogenetic evidence of this alteration, supporting the complementary role of RNA-seq in refining molecular classification beyond conventional cytogenetic findings (36, 37).

The molecular subtype distribution observed in this cohort was broadly consistent with that observed in international pediatric ALL series. Hyperdiploidy usually accounts for approximately 20–30% of pediatric B-cell ALL cases, whereas *ETV6::RUNX1* has been reported in 20–25%; both are among the most common subtypes and are generally associated with favorable outcomes when treated under contemporary protocols (38, 39). The observed frequency of Ph-like ALL in our cohort was (n = 6, 15.0%; 95% CI, 7.1–29.1%), which appears relatively high compared with several European cohorts, where frequencies of approximately 10–12% have been reported, as well as recent Latin American series from Mexico (n = 22; 9.1%), Argentina (n = 32; 9.4%), and Brazil (n = 142; 11%) (17–19). However, given the limited sample size of our cohort, this difference should be interpreted cautiously and requires confirmation in larger independent Colombian and Latin American cohorts.

The relatively high frequency of Ph-like ALL in this cohort is clinically relevant because this subtype has been associated with treatment resistance, post-induction MRD positivity, and unfavorable outcomes (40). Most Ph-like cases had a normal karyotype, illustrating the added value of RNA-seq for identifying molecular alterations that may remain undetected by conventional cytogenetic analysis. Although Ph-like ALL has been reported more frequently among Hispanic/Latino patients (41), the present study was not designed to evaluate population-level differences in subtype frequency, and genetic ancestry was not assessed. Therefore, any potential relationship with population characteristics remains speculative. Our findings are also consistent with the only available Colombian study, in which patients with Ph-like ALL showed higher leukocyte counts at diagnosis and higher MRD levels on days 15 and 33 of induction (42).

In our cohort, three patients had persistent MRD positivity through day 78 and subsequently underwent allogeneic hematopoietic stem cell transplantation; two of these patients were classified as having Ph-like ALL. Although the indication for transplantation was based on persistent high-risk MRD rather than molecular subtype alone, this observation illustrates the potential clinical relevance of integrating genomic classification with MRD-guided risk assessment. Current evidence suggests that allo-HSCT in first remission may be considered for selected patients with Ph-like ALL who have persistent MRD or other high-risk features, although its role depends on the molecular lesion, treatment response, and therapeutic protocol (43). Overall, these findings support systematic molecular characterization of Ph-like ALL in Latin American populations, particularly to identify kinase-activating lesions that may have prognostic and therapeutic implications.

The identification of less frequent molecular subtypes, such as *DUX4, MEF2D*, iAMP21, *PAX5*alt, and *KMT2A*-rearranged ALL, highlights the diagnostic resolution of RNA-seq compared with conventional cytogenetics and FISH (44–47). Subgroups such as *DUX4*-rearranged and *MEF2D*-rearranged ALL, which have distinctive transcriptomic signatures and prognostic relevance, may remain undetected without a comprehensive transcriptomic approach (44, 47–49). Similarly, rearrangements involving B-cell lineage regulatory genes, such as *PAX5*alt, are more accurately detected by RNA-seq, further supporting its value for molecular classification in ALL (50, 51).

MRD positivity at the end of induction was observed in 20.5% of patients and was predominantly detected among Ph-like cases, as well as those classified as *DUX4*, T-ALL, *MEF2D*, and hyperdiploidy. Given that MRD is one of the strongest prognostic predictors in ALL, these findings support the interpretation of treatment response in the context of molecular subtype, which may help refine relapse risk estimation and guide risk-adapted therapeutic strategies in selected patients (43, 52–54).

Integration of molecular classification modified the initial clinical risk stratification in 10% of evaluated patients, all of whom were reclassified into higher-risk categories. Specifically, two patients initially classified as intermediate risk were reclassified as high risk after the identification of Ph-like ALL and *MEF2D* subtypes. Likewise, two patients initially classified as standard risk were reassigned to higher-risk categories: one to intermediate risk associated with *PAX5*alt and one to high risk corresponding to iAMP21. This finding indicates that molecular classification does not replace conventional clinical criteria but rather complements them by identifying high-risk biological alterations that may not be evident during initial risk stratification. In the participating institutions, routine genetic characterization relies mainly on conventional karyotyping and targeted FISH assays for a limited number of recurrent abnormalities; therefore, RNA-seq provided broader molecular information that allowed a more refined risk classification. However, these reclassifications did not influence patient management because the RNA-seq results became available after the corresponding therapeutic and risk-stratification decisions had already been made. Thus, the observed reclassification reflects the potential clinical utility of comprehensive transcriptomic profiling if implemented prospectively with an appropriate turnaround time.

Among detected fusion events, *ETV6::RUNX1* (n = 6; 42.86%) and *TCF3::PBX1* (n = 3; 21.43%) were the most frequent, falling within the expected spectrum of pediatric ALL and supporting the utility of transcriptomic profiling for detecting recurrent alterations (55, 56). In addition, RNA-seq enabled the identification of rare or cryptic fusions, such as *MEF2D::BCL9*, *MEF2D::HNRNPUL1*, *SEPTIN9::ABL1*, and *KMT2A::AFF1*, highlighting its higher diagnostic resolution compared with conventional karyotyping and targeted FISH (47, 57). The detection of *MEF2D* fusion partners and ABL1-class fusions is clinically relevant because these alterations have been associated with distinctive transcriptomic signatures, adverse prognosis, and potential therapeutic implications (57–59).

Differential expression analysis showed separation between leukemic samples and non-leukemic controls. Upregulated genes were enriched for hematologic malignancy-related terms, including acute leukemia, lymphoid leukemia, and hematologic cancer, whereas downregulated genes were mainly associated with immune response, inflammation, and defense-related processes. However, these findings should be interpreted cautiously given the limited number of non-leukemic controls (n = 5) and the marked differences in cellular composition between the two groups. Therefore, the observed transcriptional differences may reflect both leukemia-associated biological programs and differences in the relative abundance of hematopoietic cell populations (60, 61). Among the upregulated genes, several were related to B-cell lineage commitment and early lymphoid differentiation, including *VPREB1, DNTT, RAG1, RAG2, PAX5, EBF1, IGLL1, BLNK, CD19, CD79A, CD79B, MME/CD10*, and *CD22*, together with leukemia-associated genes such as *FLT3, MEF2C, LEF1*, and *RUNX1*. The increased expression of B-cell lineage and early lymphoid differentiation markers is expected, at least in part, to reflect the expansion of the immature B-cell precursor population in ALL samples compared with the predominantly myeloid cellular composition of non-leukemic bone marrow. Nevertheless, the overall transcriptional pattern is consistent with biological processes previously described in ALL and supports the relevance of the expression profiles observed in this cohort, while requiring confirmation in larger studies using additional and ideally lineage-matched controls (51, 62).

Single-nucleotide variant analysis identified 36 alterations across 24 genes relevant to leukemogenesis, many of which have been previously associated with uncontrolled proliferation, impaired differentiation, and therapeutic resistance in ALL (63, 64). The most prominent variant burdens were observed in *KMT2A* and *LEF1*. *KMT2A* is involved in epigenetic regulation and *HOXA9/MEIS1* dysregulation, which has been associated with high-risk phenotypes, whereas *LEF1* is linked to the *WNT*/β-catenin pathway and has been associated with poor therapeutic response in some B-cell ALL subtypes (46, 65).

Variants were also identified in signaling genes, including *FLT3, JAK1, JAK3*, and *LCK*, supporting activation of tyrosine kinase and *JAK/STAT* pathways, characteristic features of Ph-like and *CRLF2*-rearranged ALL (40, 41, 62, 66). In addition, alterations in *IKZF1, PAX5*, and *TBL1XR1* suggest disruption of the B-cell transcriptional program, whereas variants in *NT5C2* and *PHF6* have been linked to therapeutic resistance and clonal progression (51, 67, 68). Variants in epigenetic regulators, such as *CREBBP*, *EP300*, and *KDM6A*, were also detected and have been associated with corticosteroid resistance and impaired cellular differentiation (39). The *NOTCH1* variant, identified exclusively in a T-ALL case, is consistent with its role as a recurrent driver in this lineage (69, 70). Tier 1 variants in *FLT3* c.2504A>C and *JAK3* c.1744C>T were identified in hyperdiploid cases, along with Tier 2 variants in *SMAD4*, suggesting that additional point mutations may modulate disease biology beyond the defining structural alterations (62, 71). However, because paired germline DNA was not available, the somatic origin of these RNA-seq-detected variants could not be definitively established, and these findings should therefore be interpreted as exploratory.

This study has certain limitations that should be considered when interpreting the findings. As with many studies in rare pediatric malignancies, the sample size was constrained by recruitment challenges, limiting the statistical power for analyses of less frequent molecular subtypes and subtype-specific clinical correlations. The inclusion of patients from three hospitals in Medellín, while representative of major referral centers, may not fully capture the national population; therefore, larger multicenter cohorts would help refine subtype distribution estimates. Although different therapeutic protocols were represented, treatment heterogeneity was relatively limited, as 36 of 40 patients (90%) received ALL-IC BFM 2022 and 38 of 40 (95%) were treated under ALL-IC BFM-based protocols. Nevertheless, differences in treatment regimens, risk stratification, and therapeutic intensity may have influenced the observed clinical correlations, which should therefore be interpreted as exploratory. These exploratory clinical associations require confirmation in larger and more homogeneous cohorts. The comparison between leukemic and non-leukemic bone marrow samples should be interpreted in light of differences in cellular composition between the two groups, as normal bone marrow is predominantly myeloid whereas B-ALL samples are enriched for immature B-lineage cells; future studies incorporating sorted or lineage-matched populations could further refine this analysis. While most fusions and RNA-seq-detected variants were consistent with established molecular findings, orthogonal validation using RT-PCR, qPCR, FISH, Sanger sequencing, or targeted panels was not performed for all findings, and uncommon or unexpected events should therefore be interpreted cautiously. RNA-seq also has recognized limitations for detecting copy-number alterations and confirming the somatic origin of variants in the absence of paired germline DNA.

In conclusion, this study shows that RNA-seq enables comprehensive molecular characterization of pediatric and adolescent ALL in a Colombian cohort, identifying molecular subtypes, cryptic fusions, differential expression profiles, and biologically relevant variants. The relatively high frequency of Ph-like ALL (n = 6, 15.0%; 95% CI, 7.1–29.1%) and the detection of fusions such as *MEF2D::BCL9*, *MEF2D::HNRNPUL1*, and *SEPTIN9::ABL1* highlight the diagnostic value of RNA-seq beyond conventional karyotyping and FISH. Furthermore, molecular information refined clinical risk stratification in 10% of patients, supporting the clinical relevance of this approach. These findings support the progressive incorporation of RNA-seq into the diagnostic and prognostic evaluation of ALL in Colombia and Latin America and provide a basis for future multi-omic studies in historically underrepresented populations.

## Data Availability

The original contributions presented in the study are included in the article/**Supplementary Material**. Further inquiries can be directed to the corresponding author.

## References

[1] Terwilliger T Abdul-Hay M . Acute lymphoblastic leukemia: a comprehensive review and 2017 update. Blood Cancer J. (2017) 7:e577. doi: 10.1038/bcj.2017.53 28665419 PMC5520400

[2] Hu Y Liu Y Fu J Liu Y Wang H Song Y . Global, regional, and national burden of acute lymphoblastic leukemia in children: epidemiological trends analysis from 1990 to 2021. iScience. (2024) 27:111356. doi: 10.1016/j.isci.2024.111356 39717082 PMC11664140

[3] Miranda-Filho A Piñeros M Ferlay J Soerjomataram I Monnereau A Bray F . Epidemiological patterns of leukaemia in 184 countries: a population-based study. Lancet Haematol. (2018) 5:e14–24. doi: 10.1016/S2352-3026(17)30232-6 29304322

[4] Pui CH Yang JJ Bhakta N Rodriguez-Galindo C . Global efforts toward the cure of childhood acute lymphoblastic leukaemia. Lancet Child Adolesc Health. (2018) 2:440–54. doi: 10.1016/S2352-4642(18)30066-X 30169285 PMC6467529

[5] Colunga-Pedraza PR Colunga-Pedraza JE Peña-Lozano SP Gómez-De León A Ruiz-Delgado GJ Ribeiro RC . Diagnosis and treatment of acute lymphoblastic leukemia in Latin America. Hematology. (2022) 27:971–6. doi: 10.1080/16078454.2022.2117119 36040187

[6] Siegel RL Kratzer TB Giaquinto AN Sung H Jemal A . Cancer statistics, 2025. CA Cancer J Clin. (2025) 75:10–45. doi: 10.3322/caac.21871 39817679 PMC11745215

[7] International Agency for Research on Cancer . Global Cancer Observatory (2024). Available online at: https://gco.iarc.who.int (Accessed April 13, 2025). Cancer Today – GLOBOCAN 2022 (version 1.1).

[8] de Smith AJ Jiménez-Morales S Mejía-Aranguré JM . The genetic risk of acute lymphoblastic leukemia and its implications for children of Latin American origin. Front Oncol. (2023) 13:1299355. doi: 10.3389/fonc.2023.1299355 38264740 PMC10805326

[9] Lee SHR Antillon-Klussmann F Pei D Yang W Roberts KG Li Z . Association of genetic ancestry with the molecular subtypes and prognosis of childhood acute lymphoblastic leukemia. JAMA Oncol. (2022) 8:354–63. doi: 10.1001/jamaoncol.2021.6826 35084434 PMC8796058

[10] Hu Z Kovach AE Yellapantula V Ostrow D Doan A Ji J . Transcriptome sequencing allows comprehensive genomic characterization of pediatric B-acute lymphoblastic leukemia in an academic clinical laboratory. J Mol Diagn. (2024) 26:49–60. doi: 10.1016/j.jmoldx.2023.09.013 37981088 PMC10773144

[11] Tran TH Langlois S Meloche C Caron M Saint-Onge P Rouette A . Whole-transcriptome analysis in acute lymphoblastic leukemia: a report from the DFCI ALL Consortium Protocol 16-001. Blood Adv. (2022) 6:1329–41. doi: 10.1182/bloodadvances.2021005634 34933343 PMC8864659

[12] Alaggio R Amador C Anagnostopoulos I Attygalle AD Araujo IO Berti E . The 5th edition of the World Health Organization Classification of Haematolymphoid Tumours: lymphoid neoplasms. Leukemia. (2022) 36:1720–48. doi: 10.1038/s41375-022-01620-2 35732829 PMC9214472

[13] Zaliova M Stuchly J Winkowska L Musilova A Fiser K Slamova M . Genomic landscape of pediatric B-other acute lymphoblastic leukemia in a consecutive European cohort. Haematologica. (2019) 104:1396–406. doi: 10.3324/haematol.2018.204974 30630978 PMC6601078

[14] Li JF Dai YT Lilljebjörn H Shen SH Cui BW Bai L . Transcriptional landscape of B cell precursor acute lymphoblastic leukemia based on an international study of 1,223 cases. Proc Natl Acad Sci USA. (2018) 115:E11711–20. doi: 10.1073/pnas.1814397115 30487223 PMC6294900

[15] Kroeze E Iaccarino I Kleisman MM Mondal M Beder T Khouja M . Mutational and transcriptional landscape of pediatric B-cell precursor lymphoblastic lymphoma. Blood. (2024) 144:74–83. doi: 10.1182/blood.2024023938 38588489

[16] Cree IA . The WHO classification of haematolymphoid tumours. Leukemia. (2022) 36:1701–2. doi: 10.1038/s41375-022-01625-x 35732830 PMC9252902

[17] Sánchez-Escobar N Romero-Tlalolini MD Rosas-Vargas H Jiménez-Hernández E Núñez Enríquez JC Rangel-López A . Identification of molecular subtypes of B-cell acute lymphoblastic leukemia in Mexican children by whole-transcriptome analysis. Int J Mol Sci. (2025) 26:7003. doi: 10.3390/ijms26147003 40725249 PMC12295331

[18] Ruiz MS Sosa E Avendaño D Mercado IG Lacreu ML Riccheri MC . Molecular characterization of pediatric B-cell acute lymphoblastic leukemia using integrative transcriptomics: a multicenter study in Argentina. medRxiv. (2025), 2024.09.19.24313988. doi: 10.1101/2024.09.19.2431398838621210

[19] Migita NA Jotta PY Nascimento N Vasconcelos VS Centoducatte GL Massirer KB . Classification and genetics of pediatric B-other acute lymphoblastic leukemia by targeted RNA sequencing. Blood Adv. (2023) 7:2957–71. doi: 10.1182/bloodadvances.2022009179 36848637 PMC10320209

[20] Simon A . Babraham Bioinformatics - Fastqc a Quality Control Tool for High Throughput Sequence Data (2010). Available online at: https://www.bioinformatics.babraham.ac.uk/projects/fastqc/ (Accessed September 2, 2025).

[21] Dobin A Davis CA Schlesinger F Drenkow J Zaleski C Jha S . STAR: ultrafast universal RNA-seq aligner. Bioinforma Oxf Engl. (2013) 29:15–21. doi: 10.1093/bioinformatics/bts635 23104886 PMC3530905

[22] Li H Handsaker B Wysoker A Fennell T Ruan J Homer N . The sequence alignment/map format and SAMtools. Bioinforma Oxf Engl. (2009) 25:2078–9. doi: 10.1093/bioinformatics/btp352 19505943 PMC2723002

[23] Liao Y Smyth GK Shi W . featureCounts: an efficient general purpose program for assigning sequence reads to genomic features. Bioinforma Oxf Engl. (2014) 30:923–30. doi: 10.1093/bioinformatics/btt656 24227677

[24] Posit team . Rstudio: Integrated Development Environment for R. Boston, MA: Posit Software, PBC (2025). Available online at: http://www.posit.co/ (Accessed September 2, 2025).

[25] Gu Z Hu Z Jia Z Liu J Mao A Han H . MD-ALL: an integrative platform for molecular diagnosis of B-cell acute lymphoblastic leukemia. United States: Research Square Platform LLC (2023). doi: 10.21203/rs.3.rs-2798895/v1 PMC11141650 37981856

[26] Beder T Hansen BT Hartmann AM Zimmermann J Amelunxen E Wolgast N . The gene expression classifier ALLCatchR identifies B-cell precursor ALL subtypes and underlying developmental trajectories across age. HemaSphere. (2023) 7:e939. doi: 10.1097/HS9.0000000000000939 37645423 PMC10461941

[27] Illumina . DRAGEN Secondary Analysis (RNA Pipeline) (Version 4.3.13). San Diego, CA: Illumina, Inc (2024). Available online at: https://www.illumina.com/products/by-type/informatics-products/dragen-secondary-analysis.html (Accessed September 2, 2025).

[28] Broad Institute . Genome Analysis Toolkit (Gatk) (Version 4.2.0.0). Cambridge, MA: Broad Institute (2021). Available online at: https://gatk.broadinstitute.org/ (Accessed September 2, 2025).

[29] Karchin R Pagel KA Kim R . Opencravat: Variant Annotation Tool. Baltimore, MD: Johns Hopkins University (2020). Available online at: https://opencravat.org/ (Accessed September 2, 2025).

[30] Genoox . Franklin by Genoox. Tel Aviv, Israel: Genoox Ltd (2025). Available online at: https://franklin.genoox.com (Accessed September 2, 2025).

[31] Kopanos C Tsiolkas V Kouris A Chapple CE Albarca Aguilera M Meyer R . VarSome: the human genomic variant search engine. Bioinformatics. (2019) 35:1978–80. doi: 10.1093/bioinformatics/bty897 30376034 PMC6546127

[32] The jamovi project . Jamovi (Version 2.4). Sydney, Australia: The jamovi project (2022). Available online at: https://www.jamovi.org (Accessed September 2, 2025).

[33] National Comprehensive Cancer Network (NCCN) . NCCN Clinical Practice Guidelines in Oncology (NCCN Guidelines®): Acute Lymphoblastic Leukemia. National Comprehensive Cancer Network (2024). Pennsylvania: Plymouth Meeting. Available online at: https://www.nccn.org/professionals/physician_gls/pdf/all.pdf (Accessed April 6, 2025). Version 3.2024.

[34] Inaba H Mullighan CG . Pediatric acute lymphoblastic leukemia. Haematologica. (2020) 105:2524–39. doi: 10.3324/haematol.2020.247031 33054110 PMC7604619

[35] Roberts KG Mullighan CG . Genomics in acute lymphoblastic leukaemia: insights and treatment implications. Nat Rev Clin Oncol. (2015) 12:344–57. doi: 10.1038/nrclinonc.2015.38 25781572

[36] Harvey RC Tasian SK . Clinical diagnostics and treatment strategies for Philadelphia chromosome–like acute lymphoblastic leukemia. Blood Adv. (2020) 4:218–28. doi: 10.1182/bloodadvances.2019000163 31935290 PMC6960477

[37] Mueller SB Pikman Y Tasian SK Silverman LB Harris MH Tsai HK . ETV6 fusions from insertions of exons 3-5 in pediatric hematologic Malignancies. Haematologica. (2023) 108:3471–6. doi: 10.3324/haematol.2022.282498 37381775 PMC10690910

[38] Li J Dai Y Wu L Zhang M Ouyang W Huang J . Emerging molecular subtypes and therapeutic targets in B-cell precursor acute lymphoblastic leukemia. Front Med. (2021) 15:347–71. doi: 10.1007/s11684-020-0821-6 33400146

[39] Qian M Zhang H Kham SKY Liu S Jiang C Zhao X . Whole-transcriptome sequencing identifies a distinct subtype of acute lymphoblastic leukemia with predominant genomic abnormalities of EP300 and CREBBP. Genome Res. (2017) 27:185–95. doi: 10.1101/gr.209163.116 27903646 PMC5287225

[40] Roberts KG Li Y Payne-Turner D Harvey RC Yang YL Pei D . Targetable kinase-activating lesions in Ph-like acute lymphoblastic leukemia. N Engl J Med. (2014) 371:1005–15. doi: 10.1056/NEJMoa1403088 25207766 PMC4191900

[41] Harvey RC Mullighan CG Chen IM Wharton W Mikhail FM Carroll AJ . Rearrangement of CRLF2 is associated with mutation of JAK kinases, alteration of IKZF1, Hispanic/Latino ethnicity, and a poor outcome in pediatric B-progenitor acute lymphoblastic leukemia. Blood. (2010) 115:5312–21. doi: 10.1182/blood-2009-09-245944 20139093 PMC2902132

[42] Linares Ballesteros A Yunis LK García J Aponte N Flechas J Martinez C . Philadelphia-like acute lymphoblastic leukemia: characterization in a pediatric cohort in a referral center in Colombia. Cancer Rep Hoboken NJ. (2022) 5:e1587. doi: 10.1002/cnr2.1587 34787376 PMC9124514

[43] Molica M Simio C De Fazio L Alati C Rossi M Martino M . The evolving role of hematopoietic stem cell transplantation in Philadelphia-like acute lymphoblastic leukemia: from high-risk standard to precision strategies. Cancers. (2025) 17:3237. doi: 10.3390/cancers17193237 41097763 PMC12523408

[44] Li Z Lee SHR Chin WHN Lu Y Jiang N Lim EH . Distinct clinical characteristics of DUX4- and PAX5-altered childhood B-lymphoblastic leukemia. Blood Adv. (2021) 5:5226–38. doi: 10.1182/bloodadvances.2021004895 34547766 PMC9152998

[45] Ivanov Öfverholm I Zachariadis V Taylan F Marincevic-Zuniga Y Tran AN Saft L . Overexpression of chromatin remodeling and tyrosine kinase genes in iAMP21-positive acute lymphoblastic leukemia. Leuk Lymphoma. (2020) 61:604–13. doi: 10.1080/10428194.2019.1678153 31640433

[46] Stutterheim J Van der Sluis IM Vrenken KS Pieters R Stam RW . KMT2A-rearranged acute lymphoblastic leukemia in infants: current progress and challenges. Haematologica. (2025) 110:1951–61. doi: 10.3324/haematol.2024.285642 40207717 PMC12399962

[47] Gu Z Churchman M Roberts K Li Y Liu Y Harvey RC . Genomic analyses identify recurrent MEF2D fusions in acute lymphoblastic leukaemia. Nat Commun. (2016) 7:13331. doi: 10.1038/ncomms13331 27824051 PMC5105166

[48] Liu Y Easton J Shao Y Maciaszek J Wang Z Wilkinson MR . The genomic landscape of pediatric and young adult T-lineage acute lymphoblastic leukemia. Nat Genet. (2017) 49:1211–8. doi: 10.1038/ng.3909 28671688 PMC5535770

[49] Zhang J McCastlain K Yoshihara H Xu B Chang Y Churchman ML . Deregulation of DUX4 and ERG in acute lymphoblastic leukemia. Nat Genet. (2016) 48:1481–9. doi: 10.1038/ng.3691 27776115 PMC5144107

[50] Gu Z Churchman ML Roberts KG Moore I Zhou X Nakitandwe J . PAX5-driven subtypes of B-progenitor acute lymphoblastic leukemia. Nat Genet. (2019) 51:296–307. doi: 10.1038/s41588-018-0315-5 30643249 PMC6525306

[51] Črepinšek K Klobučar N Tesovnik T Šket R Jenko Bizjan B Kovač J . PAX5 alterations in a consecutive childhood B-cell acute lymphoblastic leukemia cohort treated using the ALL IC-BFM 2009 protocol. Cancers. (2024) 16:1164. doi: 10.3390/cancers16061164 38539499 PMC10969035

[52] Eckert C von Stackelberg A Seeger K Groeneveld TWL Peters C Klingebiel T . Minimal residual disease after induction is the strongest predictor of prognosis in intermediate risk relapsed acute lymphoblastic leukaemia - long-term results of trial ALL-REZ BFM P95/96. Eur J Cancer Oxf Engl 1990. (2013) 49:1346–55. doi: 10.1016/j.ejca.2012.11.010 23265714

[53] Chiaretti S Messina M Della Starza I Piciocchi A Cafforio L Cavalli M . Philadelphia-like acute lymphoblastic leukemia is associated with minimal residual disease persistence and poor outcome. First report of the minimal residual disease-oriented GIMEMA LAL1913. Haematologica. (2021) 106:1559–68. doi: 10.3324/haematol.2020.247973 32467145 PMC8168510

[54] Yu CH Jou ST Su YH Coustan-Smith E Wu G Cheng CN . Clinical impact of minimal residual disease and genetic subtypes on the prognosis of childhood acute lymphoblastic leukemia. Cancer. (2023) 129:790–802. doi: 10.1002/cncr.34606 36537587

[55] Fuka G Kauer M Kofler R Haas OA Panzer-Grümayer R . The leukemia-specific fusion gene ETV6/RUNX1 perturbs distinct key biological functions primarily by gene repression. PloS One. (2011) 6:e26348. doi: 10.1371/journal.pone.0026348 22028862 PMC3197637

[56] Al-Kzayer LFY Saeed RM Ghali HH Tanaka M Al-Jadiry MF Faraj SA . Comprehensive genetic analyses of childhood acute leukemia in Iraq using next-generation sequencing. Transl Pediatr. (2023) 12:827–44. doi: 10.21037/tp-22-512 37305720 PMC10248931

[57] Guarnera L D’Addona M Bravo-Perez C Visconte V . KMT2A rearrangements in leukemias: Molecular aspects and therapeutic perspectives. Int J Mol Sci. (2024) 25:9023. doi: 10.3390/ijms25169023 39201709 PMC11354696

[58] Ohki K Kiyokawa N Saito Y Hirabayashi S Nakabayashi K Ichikawa H . Clinical and molecular characteristics of MEF2D fusion-positive B-cell precursor acute lymphoblastic leukemia in childhood, including a novel translocation resulting in MEF2D-HNRNPH1 gene fusion. Haematologica. (2019) 104:128–37. doi: 10.3324/haematol.2017.186320 30171027 PMC6312004

[59] Suzuki K Okuno Y Kawashima N Muramatsu H Okuno T Wang X . MEF2D-BCL9 fusion gene is associated with high-risk acute B-cell precursor lymphoblastic leukemia in adolescents. J Clin Oncol Off J Am Soc Clin Oncol. (2016) 34:3451–9. doi: 10.1200/JCO.2016.66.5547 27507882

[60] Magalhães-Gama F Kerr MWA de Araújo ND Ibiapina HNS Neves JCF Hanna FSA . Imbalance of chemokines and cytokines in the bone marrow microenvironment of children with B-cell acute lymphoblastic leukemia. J Oncol. (2021) 2021:5530650. doi: 10.1155/2021/5530650 34335758 PMC8321713

[61] Cruz-Miranda GM Olarte-Carrillo I Bárcenas-López DA Martínez-Tovar A Ramírez-Bello J Ramos-Peñafiel CO . Transcriptome analysis in Mexican adults with acute lymphoblastic leukemia. Int J Mol Sci. (2024) 25:1750. doi: 10.3390/ijms25031750 38339034 PMC10855968

[62] Gutierrez-Camino A Richer C Ouimet M Fuchs C Langlois S Khater F . Characterisation of FLT3 alterations in childhood acute lymphoblastic leukaemia. Br J Cancer. (2024) 130:317–26. doi: 10.1038/s41416-023-02511-8 38049555 PMC10803556

[63] Mullighan CG . The molecular genetic makeup of acute lymphoblastic leukemia. Hematol Am Soc Hematol Educ Program. (2012) 2012:389–96. doi: 10.1182/asheducation.V2012.1.389.3798360 23233609

[64] Roberts KG Gu Z Payne-Turner D McCastlain K Harvey RC Chen IM . High frequency and poor outcome of Philadelphia chromosome-like acute lymphoblastic leukemia in adults. J Clin Oncol Off J Am Soc Clin Oncol. (2017) 35:394–401. doi: 10.1200/JCO.2016.69.0073 27870571 PMC5455698

[65] Guo X Zhang R Liu J Li M Song C Dovat S . Characterization of LEF1 high expression and novel mutations in adult acute lymphoblastic leukemia. PloS One. (2015) 10:e0125429. doi: 10.1371/journal.pone.0125429 25942645 PMC4420493

[66] Brady SW Roberts KG Gu Z Shi L Pounds S Pei D . The genomic landscape of pediatric acute lymphoblastic leukemia. Nat Genet. (2022) 54:1376–89. doi: 10.1038/s41588-022-01159-z 36050548 PMC9700506

[67] Meyer JA Wang J Hogan LE Yang JJ Dandekar S Patel JP . Relapse-specific mutations in NT5C2 in childhood acute lymphoblastic leukemia. Nat Genet. (2013) 45:290–4. doi: 10.1038/ng.2558 23377183 PMC3681285

[68] Todd MAM Ivanochko D Picketts DJ . PHF6 degrees of separation: The multifaceted roles of a chromatin adaptor protein. Genes. (2015) 6:325–52. doi: 10.3390/genes6020325 26103525 PMC4488667

[69] Tosello V Ferrando AA . The NOTCH signaling pathway: Role in the pathogenesis of T-cell acute lymphoblastic leukemia and implication for therapy. Ther Adv Hematol. (2013) 4:199–210. doi: 10.1177/2040620712471368 23730497 PMC3666445

[70] Dai YT Zhang F Fang H Li JF Lu G Jiang L . Transcriptome-wide subtyping of pediatric and adult T cell acute lymphoblastic leukemia in an international study of 707 cases. Proc Natl Acad Sci USA. (2022) 119:e2120787119. doi: 10.1073/pnas.2120787119 35385357 PMC9169777

[71] Yin C Sandoval C Baeg GH . Identification of mutant alleles of JAK3 in pediatric patients with acute lymphoblastic leukemia. Leuk Lymphoma. (2015) 56:1502–6. doi: 10.3109/10428194.2014.957204 25146434

