# Supplementary material for: Distribution of molecular subtypes in a Colombian cohort of patients with acute lymphoblastic leukemia using whole-transcriptome analysis
Source: Front Oncol. 2026 Sep 23;16:1957558. doi: 10.3389/fonc.2026.1957558 (PMC13642327; doi:10.3389/fonc.2026.1957558)
Supplement: Supplementary file 1 [file Table3.docx]

**Supplementary Table 1**. Cytogenetic findings and NCCN risk classification of the study cohort.

The table summarizes the conventional karyotype results for each patient included in the cohort, together with the type of cytogenetic abnormality and the corresponding risk category according to NCCN criteria. Cytogenetic findings were classified as normal, numerical, structural, or complex abnormalities. Cases without cytogenetic alterations associated with a specific NCCN risk category are indicated as not applicable (N/A).

| ID del paciente | Resultado del cariotipo | Clasificación | Riesgo NCCN |
| --- | --- | --- | --- |
| LLA08 | 45,XX,-20[12] | Numérica | Estándar |
| LLA12 | 46,XY,t(9;22)(q34;q11.2)[35] | Estructural | Alto |
| LLA13 | 47,X,i(X)(q10),+8,del(9)(p13p22)[17]/46,XX[5] | Estructural | Estándar |
| LLA16 | 46,XY,?der(19)t(1;19)(q23;p13.3)[3]/46,XY[1] | Estructural | Estándar |
| LLA18 | 46,XX[17] | Normal | N/A |
| LLA20 | 45~46,XX,-?21,1dmin[cp11] | Estructural | Estándar |
| LLA23 | 46,XY(2) | Normal | N/A |
| LLA25 | 46,XY(20) | Normal | N/A |
| LLA27 | 63,XXYY,+?X,-?1,-?4,-?6,-?7,-10,-12,-13,-15,-16,+?18,-?19,-?20,+?21,1dmin(5)/46,XY(15) | Numérica | Estándar |
| LLA28 | 46,XX | Normal | N/A |
| LLA29 | 46,XY[44] | Normal | N/A |
| LLA31 | 47,XY,+11[26]/46,XY[6] | Numérica | Estándar |
| LLA34 | 46,XY(36) | Normal | N/A |
| LLA35 | 46,XY,del(9)(p13),del(13)(q12q14),der(19)t(1;19)(q23;p13.3)[7]/46,XY[10] | Compejo | Alto |
| LLA36 | 46,XX(24) | Normal | N/A |
| LLA37 | 46,XY(12) | Normal | N/A |
| LLA38 | 46,XX(31) | Normal | N/A |
| LLA40 | 46,XY,t(8;22)(q24.1;q11.2)[25]/46,XY[1] | Estructural | Alto |
| LLA41 | 45,XX,der(12)t(12;14)(p12;q11.21-14)[16]/46,XX(11) | Estructural | Estándar |
| LLA42 | 48,XX,+8,+12,der(12;18)(q10;q10)x2,t(14;14)(q11.2;q32),+22[18]/46,XX[6] | Compleja | Alto |
| LLA43 | 53,XY,+4,+75,+6,+78,+14,+21,+22[10]/46,XY[8] | Numérica | Estándar |
| LLA44 | 57-59,XXX,-1,-2,-3,-7,-12,-13,-14,-15,-16,-17,i(17)(q10),-19,-20,+21,-22[12]/46,XX[21] | Numérica | estándar |
| LLA45 | 46,XX(4) | Normal | N/A |
| LLA46 | 46,XX,t(1;2)(p36.3;p23)[26] | Estructural | Estándar |
| LLA47 | 46,XY | Normal | N/A |
| LLA48 | 46,XY | Normal | N/A |
| LLA49 | 46,XX,t(1;19)(q23;13.3)[17]/46,XX[7] | Estructural | Estándar |
| LLA50 | 46,XX,t(9;14)(p22;q11.2)[28]/46,XX[4] | Estructural | Estándar |
| LLA51 | 46,X,+X,-Y,add(19)(p13.3),trp(21)(q22)[13]/46,XY[19] | Compleja | Alto |
| LLA52 | 46,XX(4) | Normal | N/A |
| LLA53 | 46,XY | Normal | N/A |
| LLA54 | 46,XX(4) | Normal | N/A |
| LLA55 | 46,XX,+X,-15,der(17;21)(q10;q10),der(20)t(17;20)(q13.3;q11.2),+mar[33] | Compleja | Alto |
| LLA56 | 54,XX,+X,+4,+76,+10,+14,+17,+17,+21[33]/46,XX[2] | Numérica | Estándar |
| LLA57 | 45~46,XX,der(1;?)(q10;?),der(2)?t(2;17)(q34;q21),t(6;12)(q15;p13),-14,+mar1,+mar2[cp16] | Compleja | Alto |
| LLA58 | 46,XY,t(1;19)(q23;p13.3),i(9)(q10q10),der(13)t(1;13)(q25;q14)[11]/46,XY[22] | Compleja | Alto |
| LLA59 | 46,XX[17] | Normal | N/A |
| LLA60 | 59,XY,+X,+4,+б,+7,+8,+10,+12,+13,+14,+17,+21,+21,+22[18]/46,XY[16] | Numérica | Estándar |
| LLA61 | 47,XY,t(2;14)(p11.2;q32),del(12)(p12),+21[17]/46,XY[2) | Estructural | Estándar |
| LLA62 | 46, XX, del(6) (q13q21)[18]/46,XX[19] | Estructural | Estándar |

**Supplementary Figure 1. Gene Ontology enrichment analysis of genes downregulated in ALL.**
The plot shows enriched Biological Process Gene Ontology terms among genes downregulated in acute lymphoblastic leukemia samples compared with non-leukemic bone marrow controls. The x-axis represents the enrichment signal, bubble size indicates the number of genes associated with each term, and color represents the false discovery rate (FDR). The most enriched processes were related to inflammatory response, immune system processes, defense response, and response to biotic stimuli.

**Supplementary Figure 2. Disease-gene association enrichment of genes upregulated in ALL.**
Enriched disease-gene association terms among genes upregulated in acute lymphoblastic leukemia samples were mainly related to hematologic malignancies, including acute leukemia, lymphoid leukemia, hematologic cancer, lymphoma, and leukemia. Bubble size represents gene count, and color indicates the false discovery rate (FDR).
